# Rhinitis medicomentosa and substance addiction

**DOI:** 10.1007/s00405-024-08723-9

**Published:** 2024-05-14

**Authors:** Mehmet Birinci, Dogukan Ozdemir, Meltem Pusuroglu, Ömer Sevim, Tuğba Yemiş, Seda Nur Cihan, Esra Yılmaz, Metin Çeliker, Özlem Çelebi Erdivanlı

**Affiliations:** 1 Faculty of Medicine, Department of Otorhinolaryngology, Recep Tayyip Erdogan University, Rize, Turkey; 2 Faculty of Medicine, Department of Otorhinolaryngology, Samsun University, Samsun, Turkey; 3 Faculty of Medicine, Department of Psychiatry, Recep Tayyip Erdogan University, Rize, Turkey

**Keywords:** Rhinitis medicomentosa, Nasal decongestant, Substance abuse proclivity, Depression

## Abstract

**Background:**

Rhinitis medicomentosa (RM) is a form of non-allergic rhinitis caused by the use of nasal decongestants for longer than the recommended duration. Because of this problem of use, addiction to the drug occurs in individuals. In our study, we aimed to evaluate the susceptibility of RM patients to substance addiction.

**Methods:**

The study was planned as a prospective, multicentric study between September 2022 and September 2023. Patients diagnosed with RM were included in the study. Beck depression scale, Drug use disorders identification test, Substance Abuse Proclivity Scale were applied to the patients participating in the study. The research data were analyzed electronically with SPSS program version 25.

**Results:**

The study included 86 patients with an average age of 31 years. The average duration of medication use was 22 months. Age, gender, duration of nasal congestion, duration of drug use and smoking were not independent predictors for depression and substance use tendency.

**Conclusion:**

The relationship between RM and addictive substances is not clear. The tendency to use drugs did not increase in RM patients. In the light of these data, we think that there is no need for a practice other than routine functioning in the use of drugs and similar substances that are likely to cause addiction in RM patients.

## Introductıon

Nasal decongestants are frequently preferred drugs in acute upper respiratory tract infections. Since they rapidly resolve nasal obstruction and are available without a prescription, they may be used more than necessary [1].

Rhinitis medicomentosa (RM) is a rhinitis condition caused by prolonged use of nasal decongestants [2]. It was first described by Fox [3] and Feinberg first mentioned rebound decongestion [4]. In 1946, Lake used the term rhinitis medicomentosa [5]. RM is defined as one of the phenotypes of non-allergic rhinitis (NAR). In addition to those who consider it as a part of the drug-induced rhinitis group, there are authors who think that it is a different phenotype due to the different use and mechanism of action of the drug [6].

Psychiatric predispositions in RM patients, which have been demonstrated with limited data in previous studies, were planned to be examined from a different perspective in our study. The aim of this study is to determine the predisposition to substance abuse in patients who use nasal decongestants for a long period of time despite the prescribed duration of treatment. In the light of the data of this study, we aimed to answer the question of whether there is a group that should be particularly questioned in patients who are recommended decongestants.

## Materials and methods

Patients diagnosed with rhinitis medicomentosa who used topical nasal decongestants for at least 2 months between September 2022 and September 2023 at Recep Tayyip Erdoğan University Training and Research Hospital and Samsun Training and Research Hospital were prospectively evaluated. Patients were analyzed in terms of demographic characteristics, decongestant content and duration of decongestant use. All patients underwent a detailed otolaryngologic examination and psychiatric evaluation. Beck Depression Scale, Drug Use Disorders Identification Test, and Substance Abuse Proclivity Scale questionnaires were applied to the patients who participated in the study. Ethics committee approval was obtained from Recep Tayyip Erdogan University Faculty of Medicine Non-Interventional Clinical Research Ethics Committee before the study (Decision no: 2023/204) and the study was conducted in accordance with the Helsinki declaration. The research data were analyzed electronically with SPSS program version 25. Descriptive statistics of the data were expressed as mean ± standard deviation, median (minimum–maximum), number and percentage. Normality of continuous data was analyzed using the Kolmogorov–Smirnov test, skewness kurtosis values and histograms. Substance use tendency and depression were categorically defined as present or absent. A logistic regression model was established with the dependent variable of substance use and the independent variables of age, gender (female), duration of nasal congestion, duration of drug use, and smoking (yes). Likewise, a logistic regression model was established with the dependent variable of depression and the independent variables of age, gender (female), duration of nasal congestion, duration of drug use, and smoking (yes). Statistical significance value was taken as p < 0.05.

## Results

The study included 86 patients. The youngest patient was 18 years old and the oldest was 57 years old with a mean age of 31.15 ± 9.645 years. 38 (44.2%) were female and 48 (55.8%) were male; 9 (10.5%) were primary school graduates, 7 (8.1%) were middle school graduates, 25 (29.1%) were high school graduates, and 45 (52.3%) were university graduates. 23 (26.7%) are unemployed, 18 (20.9%) are civil servants, 9 (10.5%) are retired and 12 (14%) work in other occupational groups. 52 (60.5%) are married, 24 (27.9%) are single, 10 (11.6%) are divorced. 44 (51.2%) were smokers. 57% of the patients reported that they used xylometazoline and 38% used oxymetazoline. 2 patients reported using whichever active ingredient they could find, and 2 patients were using products containing xylometazoline dexpentanol combination. The mean duration of nasal congestion was 9.79 ± 6.998 month and the mean duration of medication use was 22.79 ± 33.475 month (Table 1). Risk factors such as age, gender, duration of nasal congestion, duration of drug use and smoking for the prediction of depression and substance use tendency were analyzed. None of the variables were found to be as independent predictors of depression (Table 2) and substance use tendency (Table 3) (p > 0.05).

**Table 1:** Demographic characteristics for rhinitis medicamentosa patient

	Mean ± SD	Med (min–max)
Age	31.15 ± 9.645	29(18–57)
Duration of nasal obstruction (month)	9.79 ± 6.998	8(1–40)
Duration of drug usage (month)	22.79 ± 33.475	12(2–240)

	n	%
Gender		
Male	48	55.8
Female	38	44.2
Education		
Elementary school	9	10.5
Middle school	7	8.1
High school	25	29.1
University	45	52.3
Occupation		
Unemployed	23	26.7
Worker	18	20.9
Civil servant	24	27.9
Retired	9	10.5
Other	12	14.0
Marital status		
Married	52	60.5
Single	24	27.9
Divorced	10	11.6
Smoker		
No	44	51.2
Yes	42	48.8
Total	86	100

**Table 2:** Logistic regression model for depression and related factors

	Univariate		Multivariate	
	OR (%95 CI)	p	OR (%95 CI)	p
Age	0.953	0.998(0.936–1.064)	1.015(0.952–1.082)	0.649
Gender(Female)	0.293	1.942(0.564–6.691)	2.237(0.583–8.587)	0.241
Duration of Nasal obstruction	0.299	0.939(0.835–1.057)	0.924(0.809–1.056)	0.248
Duration of Drug Usage	0.370	0.981(0.940–1.023)	0.981(0.933–1.031)	0.446
Smoker(Yes)	0.480	1.560(0.454–5.364)	1.853(0.490–7.009)	0.363

Cox&Snell R^2^ = 0.051; Nagelkerke R^2^ = 0.092; Hosmer and Lemeshow Chi Square = 7.654, p = 0.481

**Table 3:** Logistic regression model for substance use tendency and related factors

	Univariate		Multivariate	
	OR (% 95 CI)	p	OR (% 95 CI)	p
Age	0.980(0.937–1.025)	0.370	0.987(0.940–1.037)	0.611
Gender (female)	0.980(0.937–1.025)	0.370	0.755(0.301–1.891)	0.548
Duration of nasal obstruction	0.937(0.876–1.003)	0.062	0.939(0.873–1.011)	0.094
Duration of drug usage	0.995(0.982–1.008)	0.427	0.999(0.983–1.015)	0.889
Smoker (yes)	1.354(0.573–3.201)	0.490	1.403(0.572–3.442)	0.460

Cox&Snell R^2^ = 0.058; Nagelkerke R^2^ = 0.078; Hosmer and Lemeshow Chi Square = 9.034, p = 0.396

## Discussion

In this study, it was aimed to evaluate the substance use tendencies of RM patients, which is considered as a type of addiction. It is the first prospectively planned study on this subject in the literature. It is also the first research questioning all types of addiction in this patient group. As a result of the study, depression, drug use and tendency were not detected in RM patients.

In a prevalence study from the Netherlands, the prevalence of NAR was found to be 27%. In order of frequency, RM ranks 2nd and is reported to account for 14% of NAR patients [6].

Prolonged use of nasal decongestants causes vasoconstriction and ischemia of the mucosa, resulting in interstitial edema [7]. As a result of prolonged α-2 receptor stimulation, the receptors become down-regulated. With this effect, the vasoconstriction property of the mucosal sinusoidal venous plexus decreases and a relative expansion is observed. On the other hand, as the receptors become resistant to nasal decongestants, patients need to increase the dose. Since this dose increase causes the person to get rid of nasal congestion, it increases the rewarding effect on the person. This represents a form of withdrawal in some people and may lead to addiction [8]. This mechanism is similar to the mechanism of substance addiction.

Smoking is known to have several effects on the nasal mucosa. Increased epithelial thickness, increased number of goblet cells, presence of oedema and congestion in the nasal mucosa are some of these effects [9]. Another effect is an increase in nasal epithelial dysfunction. It has also been shown that this adverse effect increases with the duration of exposure [10]. Smoking rates in RM patients have been found to be higher in studies. It was even found to be 10 times higher in percentage compared to control groups. In the same study, the rate of smoking in the RM group was found to be 53% and in our study, the rate of smoking was 51.2% [8, 11]. This is higher than the cigarette smoking rates of the normal population. In previous studies, the relationship between smoking and chronic rhinosinusitis has been clearly demonstrated [12]. The reason for the high rates of smoking in our and previous studies may be the desire to eliminate the obstruction caused by these sinonasal diseases with nasal decongestants. These data suggest that there may be a relationship between nicotine addiction and decongestant addiction. Future studies are obviously needed for this.

The presence and severity of nasal symptoms were not associated with the risk of developing RM in a study from Italy. Sleep disorders have been shown to cause overuse of decongestants. The same study reported that a history of psychiatric disorders, especially anxiety, affected both the development of RM and drug discontinuation in RM patients. In the same study, they found that patients with a pathologic Hamilton anxiety score had a lower mean daily medication use, a lower mean time to discontinue medication and a lower likelihood of discontinuing medication. Nevertheless, it is reported that the duration of medication use is not affected by the duration of use [8]. The age, gender, duration of nasal obstruction, duration of medication use and smoking were not evaluated as risk factors for depression and substance abuse tendency in our study. In contrast to previous literature, there was no association between depression and RM. Similarly, no tendency to substance abuse was found in these patients. There is no data to support the belief that more care and information should be taken when prescribing these drugs, especially in patients with a psychiatric history, than in the normal population.

Depression is a major mental illness with a prevalence of 5% worldwide, characterised by symptoms such as demoralisation, apathy and lack of enjoyment of life. It is an important public health problem because of the loss of functioning it causes in patients. Female gender, young age, low socio-economic status, trauma and chronic illness are risk factors for the development of depression. In the course of chronic diseases, the treatment burden on patients and the decrease in their quality of life lead to a predisposition to depression [13–15]. Indeed, patients with rhinitis medicamentosa are individuals who suffer from nasal congestion for an extended period of time. The presence of nasal congestion and other symptoms may have a negative impact on the quality of life and may predispose to mental illnesses such as depression [16]. Although factors such as age and gender were not found to be related to depression in our study, 15.7% of our sample had scores above the cut-off point of the Beck Depression Scale. This is a value above the general depression rates in the society. Although the patients were not diagnosed with depression with a clinical interview, the scores obtained from the beck depression scale suggest that depression symptoms may be higher in RM patients than in the general population. Further studies with larger samples are needed in this subject.

In 2019, a retrospective study by Patel et al. found that opioid dependence was higher in patients with rhinitis medicomentosa than in patients with rhinitis who did not use decongestants. Despite the exclusion of patients with psychiatric history, increased opioid use in RM patients is thought to support the psychological component here. These data were shown as evidence of an addictive component of RM. However, it is also reported that half of the patients in the studied group underwent surgery and opioids were prescribed to these patients after surgery [17]. On the other hand, in our study, patients were evaluated in terms of substance use, but no significant results were obtained. We believe that the difference here is that opioids are not routinely used in postoperative pain control by the clinics conducting the study. The difference in the study by Patel et al. is due to the high rate of opioid use. Literature also shows that opioid addiction is high in postoperative follow-ups where opioid use is high [18].

There are some limitations of our study. Number of cases and the fact that our study was conducted in only 2 centers is one of them. Improved results can be obtained from studies with more clinics and cases. In addition, the fact that none of the patients in our patient group had substance abuse is another limitation.

Although the mechanism of RM is not clearly defined, it is considered as a form of addiction. The relationship with other addictive substances is not clear. There is no tendency to drug use in RM patients. With these data, there is no need for a different practice than the normal population in the use of drugs and similar substances that are likely to cause addiction in RM patients.

## Data Availability

The data that support the findings of this study are available on request from the corresponding author, MB. The data are not publicly available due to [restrictions e.g. their containing information that could compromise the privacy of research participants].
